# Effects of Salinity Stress at Reproductive Growth Stage on Rice (*Oryza sativa* L.) Composition, Starch Structure, and Physicochemical Properties

**DOI:** 10.3389/fnut.2022.926217

**Published:** 2022-06-29

**Authors:** Dongping Yao, Jun Wu, Qiuhong Luo, Dongmeng Zhang, Wen Zhuang, Gui Xiao, Qiyun Deng, Bin Bai

**Affiliations:** ^1^State Key Laboratory of Hybrid Rice, Hunan Hybrid Rice Research Center, Changsha, China; ^2^College of Plant Science and Technology, Hunan Biological and Electromechanical Polytechnic, Changsha, China; ^3^National Center of Technology Innovation for Saline-Alkali Tolerant Rice in Sanya, Sanya, China; ^4^College of Agronomy, Hunan Agricultural University, Changsha, China; ^5^Bio-Rice (Hunan) Co. Ltd., Changsha, China

**Keywords:** rice composition, salinity stress, reproductive growth stage, starch structure, starch physicochemical property

## Abstract

This study aimed to investigate the changes in polished rice composition, starch structure, and physicochemical properties from three rice cultivars treated with medium and high salinity stress at the reproductive growth stage. The results showed that salt stress led to poor milling and appearance quality, higher total starch content, protein content, higher proportion of the medium, and long chains of amylopectin, as well as gelatinization temperature (GT) but lower amylose content and lower proportion of the short chain of amylopectin. Compared with salt-sensitive cultivars, the salt-tolerant cultivars exhibited lower GT and gelatinization enthalpy, better pasting properties, and more stable crystal structure; therefore, their eating and cooking quality (ECQ) was less affected. The above results imply that salt stress at the reproductive growth stage can degrade ECQ and can slightly increase the pasting property of starch from salt-tolerant rice cultivar.

## Introduction

Rice (*Oryza sativa* L.) is one of the most important food crops providing more than 20% of the world's total dietary energy. It is grown in many parts of the world, especially in Asia where almost 90% of rice is cultivated (1). Rice grain is mainly composed of carbohydrates, most predominantly starch which accounts for 50–90% of rice grain dry weight. The changes in starch-related structure and physicochemical properties affect directly rice quality, especially eating and cooking quality (ECQ) (2). ECQ is usually evaluated by the amylose content, amylopectin chain length distribution, gelatinization temperature (GT), and pasting property (3). Yield stability is a primary agronomic concern where rice is critical to food security, while ECQ is an important factor to determine the market acceptance and price of rice (4). Both yield stability and ECQ are affected easily by environmental factors, and there is an increasing concern about both specific effects of environmental stress. Salinity stress is a major environmental factor limiting plant growth and crop production. Nearly 20% of arable land and half of the irrigated areas of the world are under salt stress, which leads to significant economic losses of food crops (5). Salinity stress, especially a high concentration of NaCl, is mainly caused by osmotic inhibition and ionic toxicity (6), which ultimately inhibits crop photosynthesis, decreases yield, and has a great influence on the accumulation, structure, and physicochemical properties of starch in cereal crops (7).

Among all the crops, rice is the first choice of grain crops for improving the quality and efficiency of saline–alkali land, but most rice cultivars are sensitive to salinity stress with the threshold limit of electrical conductivity (EC) around 3 dS/m (1). Soils with exceeding EC of 4 and 8 dS/m are considered moderate and high salt stress, respectively (8–10), and the salinity level of more than 5 dS/m will cause a significant reduction in terms of rice yield and quality (11). Being a moderately salt-sensitive crop, the responses of rice to salt stress are complex and its sensitivity varies from species to species, growth and development stages, and stress duration (12). Generally, the seedling stage and reproductive growth stage are the most sensitive to salt stress, and there is no necessary correlation between salt tolerances at different growth stages (13). The reproductive growth stage has the greatest impact on yield and quality (14). Panicle sterility is considered to be a major threat under salt stress during the rice reproductive stage (5). In terms of milling and appearance quality, most of the studies reported that salt stress had the greatest effect on the head rice rate and chalkiness, followed by the polished rice rate, the brown rice rate, and the grain length/width ratio (15).

A few studies on the effects of salt stress on physicochemical properties of rice starch have been reported. However, the results of the studies on the response of rice quality to salt stress are not consistent due to the differences in terms of treatments and tested rice cultivars. Aref and Rad (16) reported that salt stress significantly decreased starch synthase activity, which led to the reduction of starch accumulation. In addition, Rao et al. (17) proposed that 8 dS/m or higher salinity levels reduced amylose content. In contrast, Thitisaksakul et al. (14) found that salinity with EC of 2 or 4 dS/m increased amylose content during seedling and flowering stages. Some studies suggested that amylose content and pasting property being measured by Rapid Visco Analyser (RVA) were closely related to the salt concentration of treatment. Under low salt concentration, the viscosity of rice starch was generally higher than that of the control, while amylose content was lower than that of the control. Under high salt concentration, the amylose content of rice increased, while the viscosity decreased significantly compared with the control (15, 18). The effects of salt stress on physicochemical properties of rice starch need further study and systematic analysis. Therefore, the study was carried out to elucidate the effects of salinity stress on rice composition, starch structure, and physicochemical properties, in order to systematically evaluate the quality, especially ECQ level of salt-tolerant rice cultivars under salt stress.

## Materials and Methods

### Plant Materials and Planting Conditions

The salt stress experiment was carried out in the rainproof canopy of State Key Laboratory of Hybrid Rice, Hunan Hybrid Rice Research Center in Changsha City, Hunan Province, China, in 2020. The roof of the canopy was covered with white transparent plastic film with light transmittance of more than 90% and surrounded by barbed wire to ensure the circulation of natural air. There were three soil culture ponds in the rainproof canopy, each of which was 4.7 m long, 1.3 m wide, and 0.9 m high of soil.

Three promising rice cultivars were selected as plant materials for this experiment, based on their different response to salinity stress, namely, Xiang liangyou 900 (salt-tolerant cultivar), Y liangyou 900 (salt-tolerant cultivar), and IR64 (salt-sensitive cultivar) which were abbreviated as XLY900, YLY900, and IR64, respectively (Supplementary Table S1). These three cultivars were provided by Hunan Hybrid Rice Research Centre and showed relatively large differences in response to salt stress, based on our preliminary experiment on the evaluation of the salt tolerance of 28 rice cultivars by salinity treatment of 12 and 5 dS/m in the experimental base of National Center of Technology Innovation for Saline-Alkali Tolerant Rice in Sanya City of China. As the growth period of YLY900 is 2 days earlier than that of XLY900, while the growth period of IR64 is 5 days earlier than that of XLY900, the full and fresh seeds were sown on 30 April, 3 May, and 6 May, respectively, for XLY900, YLY900, and IR64 to adjust the growth period of three cultivars, thus ensuring that the growth and development stages of each cultivar were consistent. When the seedlings grew to the three-leaf stage, seedlings with the same growth were selected and transplanted to soil culture ponds. Water and fertilizer management was the same as in the field. The total nitrogen application was 180 kg·hm^−2^, and the ratio of nitrogen, phosphorus, and potassium was 1.0:0.6:1.1. In the base fertilizer, N and K accounted for 30 and 40% of the total N and K application, respectively, and all P fertilizer was used as the base fertilizer. In tillering fertilizer, N and K accounted for 40 and 20% of the total N and K application, and the remaining N and K fertilizers were used as panicle fertilizer.

### Salinity Stress Treatments

Salt stress was designed to achieve EC values of 12 and 5 dS/m by adding 137 and 51 mM NaCl into two soil culture ponds at reproductive (R) physiological stages (R2 stage until maturity) of rice (19, 20). Another soil culture pond was used as control without the addition of NaCl and its EC was maintained at EC 0–1 dS/m. Three soil ponds constituted three salt ponds, and each salt pond was planted alternately in rows of XLY900, YLY900, and IR64 (Supplementary Figure S1). Digital Hand-Held “Pocket” Salt Meter (PAL-SALT, ATAGO, Japan) was used to monitor the salt concentration of the water layer every day and to adjust in a timely manner the salt concentration of the water layer being changed by evaporation. The water was cut off 7 days before the harvest. The field sowing, transplanting, management, and sampling methods were the same for all cultivars of the three batches of rice used in the experiment (2).

### Measure Items and Methods

#### Determination of Milling and Appearance Quality

Brown rice rate, polished rice rate, head milled rice rate, the ratio of grain length to width, and chalkiness degree were determined according to the agricultural industry standards NY/T 83-2017 and NY/T 2334-2013 of the People's Republic of China to evaluate grain milling and appearance quality. To determine the milling quality, 140 g of rice was dehulled using a huller (THU35C, SATAKE, Japan) and weighed. The brown rice rate was then calculated. It was then followed by the processing of 100 g of brown rice into milled rice using a milling machine (LTJM-2099, Zhengzhou Zhonggu Machinery Equipment Co., Ltd, Zhengzhou, China) and weighed to calculate the polished rice rate. The Image Analysis Software SC-E (Hangzhou Wanshen Detection Technology Co., Ltd, Hangzhou, China) was used to obtain the head milled rice rate. In contrast, to determine the appearance quality, scanning images of milled rice were obtained by a scanner (MICROTEK ScanMaker i800 plus, Shanghai Zhongjing Technology Co., Ltd, Shanghai, China). The Image Analysis Software SC-E was then used to analyze the scanned images of the head milled rice to identify the number and area of chalky grains and to calculate the chalkiness, grain length, grain width, and length–width ratio.

#### Determination of Polished Rice Composition, Starch Isolation, and Starch Physicochemical Indexes

Rice starch isolation, gelatinization property, pasting property, and X-ray diffraction (XRD) measurement were determined to evaluate the effects of physicochemical properties of rice starch by salt stress using the methods described by Yao et al. (2). Total starch content was determined using the total starch detection kit (Megazyme, K-TSTA).

The amylose content was determined according to the agricultural industry standard NY/T 2639 of the People's Republic of China. To determine the amylose content, 10 g of milled rice was accurately weighed and pulverized using a mill. After sifting through a 0.25-mm sieve, the sample was poured into a paper bag and placed under the same conditions for 2 days with amylose reference samples (purchased from China National Rice Research Institute, containing sticky rice, low, medium, and high amylose reference samples) to balance water content. The samples to be tested and reference samples were weighed (0.1 g ± 0.2 mg) and placed in a 100-ml volumetric flask. A volume of 1.0 ml of 95% ethanol was added to moisten and disperse the samples, 9.0 ml sodium hydroxide solution (1.0 mol/L) was added, and then the volumetric flask was placed in a boiling water bath for 10 min. After cooling to room temperature, distilled water was added for constant volume to serve as an amylose reference solution and a sample test solution. At the same time, 9.0 ml sodium hydroxide solution (1.0 mol/L) and 91.0 ml distilled water were added to a 100-ml volumetric flask to serve as a blank solution. A volume of 2.5 ml amylose reference solution and sample solution to be tested was absorbed in a 50-ml volumetric flask and then added with about 25 ml water, 0.5 ml acetic acid solution (1 mol/L), and 1.0 ml iodine solution (weighed 2.0 g I_2_ and 20 g kI and dissolved them in a small amount of water, constant volume to 1,000 ml). Water was then added to a constant volume and the mixture was allowed to rest for 10 min. Finally, a spectrophotometer (SP-722, Shanghai Spectral Instrument Co., Ltd., Shanghai, China) was used and set to zero with the blank solution, while the absorbance value of the reference standard solution was measured at the wavelength of 620 nm. The standard curve was drawn with the absorbance value as the ordinate and amylose content as the abscissa. The absorbance value of the solution to be tested was determined at the wavelength of 620 nm, and its amylose content was converted by the standard curve. Two parallel samples were taken from each sample for determination, and the arithmetic mean value was taken as the result.

The protein content was determined according to the national standard GB 5009.5-2016 of the People's Republic of China. An amount of 0.2 g (accurate to 0.001 g) of powder samples was weighed in the digestion tube (2.25 g K_2_SO_4_ and 0.25 g CuSO_4_·5H_2_O), 5 ml of concentrated sulfuric acid was added as a catalyst, and the tube was gently shaken to soak the sample and placed on a Digestor (Labtec DT208 Digestor, FOSS, Denmark) for digestion (at the same time, blank test being done). When the temperature of the Digestor reached 420°C, the digestion continued for 60 min. The digestion tube was cooled to room temperature, added 5 ml of distilled water, and connected to an Automatic Kjeltic Nitrogen Analyzer (Kjeltic 8400, FOSS, Denmark) that had been added sodium hydroxide solution, hydrochloric acid or sulfuric acid standard solution, and boric acid solution containing mixed indicator. The determination procedure of the Kjeldahl apparatus includes the addition of liquid automatically, followed by distillation, titration, and calculation. The protein content of the sample was recorded after titration. Two parallel samples were taken from each sample for determination, and the arithmetic mean value was taken as the result.

#### Amylopectin Chain Length and Granule Size Distribution

Starch chain length distribution was determined by the ICS5000 ion chromatography system (ICS-5000, Thermo Fisher Scientific, Sunnyvale, USA) as performed by Nishi et al. (21) with a minor modification. The Dionex™ CarboPac™ PA200 (3.0 × 250 mm; Thermo Fisher Scientific, Sunnyvale, United States) ionic column was used according to the properties of the glucose chain. Mobile phase A (aqueous solution), mobile phase B (100 mM NaOH and 1 M NaAC), and mobile phase C (100 mM NaOH) were used, with the flow rate controlled at 0.4 ml/min, and the column temperature at 30°C.

The starch granule size distribution was measured using a Malvern laser particle size analyzer (Mastersizer 3000, Malvern Instruments Ltd., UK). Starch powder (100 mg) was weighed and dispersed in 1 ml of absolute ethanol, which was then stirred at 2,000 rpm. The blended samples were placed in the Mastersizer 3000 for determination. The results were analyzed using the Mastersizer 3000 software. According to the method described by Xiong et al. (22), the volume, surface area, and number distribution of the measured starch granules were divided into two types, namely, small and medium starch granules (starch granule diameter *d* < 10 μm) and large starch granules (*d* > 10 μm).

### Statistical Analysis

All parameters shown in the tables and figures used in this article represent the mean values of the experimental data obtained from triplicate tests for all cultivars sown at three salt ponds. The analysis of all data was performed using the SPSS 24.0 statistical software program (IBM Corporation, Armonk, New York). One-way analysis of variance and Tukey's tests were used to determine whether statistically significant differences (*P* < 0.05) existed among the means.

## Results

### Rice Milling and Appearance Quality

Except for the polished rice rate of IR64, the brown rice rate, polished rice rate, and head milled rice rate of all cultivars were not affected by medium salt stress but decreased significantly under high salt concentration (Supplementary Table S2). Chalkiness was the most sensitive index to salinity stress among all milling and appearance quality. Salinity stress at the reproductive growth stage significantly increased chalkiness degree and chalkiness grain rate, and the changes were more significant under high salt stress. For salt-tolerant cultivars under medium and high salt concentrations, chalkiness of XLY900 increased by 385 and 628%, respectively, and chalkiness grain rate increased by 306 and 536%, respectively. However, for YLY900 at medium and high salt concentrations, the chalkiness increased by 715 and 883%, and the chalkiness grain rate increased by 286 and 384%, respectively. The difference in the effect between medium and high salt levels in XLY900 was greater than that in YLY900, which might be attributed to the different response mechanisms between these two cultivars. The chalkiness of salt-sensitive cultivar was most seriously affected by salt stress and increased obviously at medium salt level, but there was no significant difference between medium salt level and high salt level, indicating that the structure of salt-sensitive cultivar had been damaged by medium salinity level. The length–width ratio was the least sensitive to salt stress for these three cultivars.

### Chemical Composition of Polished Rice

Salt stress significantly decreased the amylose content of YLY900 and IR64, but the degree of decline was consistent between medium and high salt stress (Table 1). Only the total starch content of XLY900 and YLY900 significantly increased under salt stress. After salt stress treatment, the protein content of the three cultivars showed the same trend, which only increased significantly under high salt stress, while the medium salt concentration had no significant change, indicating that the protein content was not sensitive to medium salt stress, and only the higher salt concentration could lead to the change of protein content.

**Table 1:** Effects of salt stress on the chemical composition of polished rice.

**Cultivars**	**EC (dS/m)**	**Total starch content (%)**	**Protein content (%)**	**Amylose content (%)**
XLY900	12	80.18 ± 0.79a	12.44 ± 0.14a	20.42 ± 0.09a
	5	78.43 ± 2.21b	11.16 ± 0.46b	20.03 ± 0.43a
	0–1	78.94 ± 1.74b	10.74 ± 0.04b	20.10 ± 0.32a
YLY900	12	80.35 ± 0.73a	10.16 ± 0.05a	12.23 ± 0.48b
	5	79.17 ± 6.51a	9.94 ± 0.17b	12.25 ± 0.10b
	0–1	77.73 ± 1.99b	9.66 ± 0.15b	13.00 ± 0.08a
IR64	12	76.62 ± 1.14a	12.90 ± 0.11a	15.15 ± 0.34b
	5	75.29 ± 1.07a	12.03 ± 0.43b	15.48 ± 0.79b
	0–1	76.80 ± 4.08a	11.67 ± 0.21b	16.00 ± 0.22a

*Data are shown as the mean ± standard error of triplicate measurements. Different letters following the standard deviation denote significant differences (p < 0.05). XLY900, Xiang liangyou 900; YLY900, Y liangyou 900; EC, electrical conductivity*.

### Amylopectin Chain Length Distribution

**Figure 1:**
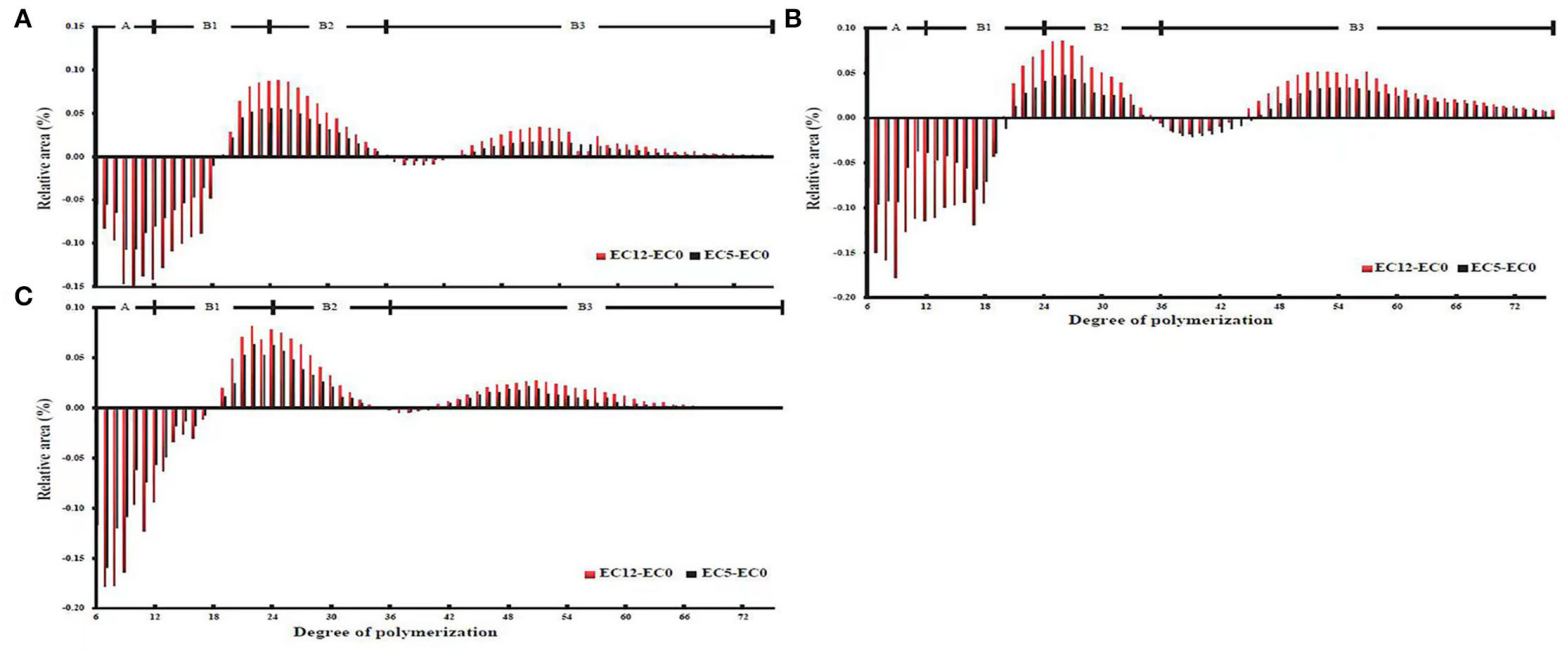
Starch chain distribution of Xiang liangyou 900 **(A)**, Y liangyou 900 **(B)**, IR64 **(C)** under salt stress, and the control. The branch chain length was categorized into four classes: a class (6 ≤ DP ≤ 12), B1 class (12 < DP ≤ 24), B2 (24 < DP ≤ 36), and B3 class (36 < DP ≤ 76). EC12, EC5, and EC0 represent that the electrical conductivity is 12, 5, and 0–1 dS/m, respectively.

As for the chain length distribution of amylopectin, it can be seen from the general trend that the three cultivars had similar changes, showing that the short chain with low polymerization degree decreased, while the middle and long chains with high polymerization degree increased, which promoted the formation of more perfect crystals (Figure 1) (23). According to previous studies, amylopectin chain was divided into four types, namely, A chain (6 ≤ DP ≤ 12, short chain), B1 chain (13 ≤ DP ≤ 24, medium chain), B2 chain (25 ≤ DP ≤ 36, medium and long chains), and B3 chain (DP ≥ 37, long chain) (24). As shown in Figure 1, the three cultivars showed the same change trend, i.e., a chain of amylopectin decreased significantly, while medium and long chains B2 and B3 increased obviously under the increasing salt stress. The proportion of B1 chain in all three cultivars first decreased and then increased, but the overall trends of B1 chain decreased in salt-tolerant cultivars, while it increased in the salt-sensitive cultivar. Compared with the salt-tolerant cultivars XLY900 and YLY900, the salt-sensitive cultivar IR64 showed a greater decrease in the A chain, but a lower degree of increase in the medium and long chains B2 and B3. The salt-tolerant cultivars with more medium and long chains of amylopectin promoted the formation of the double helix, which was conducive to the crystallization of starch granules (25).

### Starch Granule Size Distribution

For the salt-tolerant cultivars YLY900 and XLY900, with the increase of salt stress, the proportion of surface area, volume, and number of small and medium starch granules decreased first and then increased, while large starch granules increased first and then decreased (Table 2). Salt stress affected YLY900 more than XLY900, which might be related to the higher proportion of small and medium starch granules in XLY900. For salt-sensitive cultivar IR64, the number, volume, and surface area proportion of small and medium starch granules showed a significant decrease, while the large starch granules showed an increase. Except for the number proportion of starch granules, there was no significant change between high and medium salt stress.

**Table 2:** Effects of salt stress on starch granule size distribution.

**Cultivars**	**EC (dS/m)**	**Relative crystallinity (%)**	**Crystal pattern**	**Volume percentage (%)**		**Surface area percentage (%)**		**Number percentage (%)**	
				**d <10 μm**	**d > *10 μ*m**	**d > *10 μ*m**	**d > *10 μ*m**	**d > *10 μ*m**	**d > *10 μ*m**
XLY900	12	35.45	A	34.62 ± 0.41a	65.38 ± 0.41c	57.68 ± 0.37a	42.32 ± 0.37c	89.07 ± 0.17a	10.93 ± 0.17b
	5	46.76	A	27.32 ± 0.69c	72.71 ± 0.69a	47.90 ± 0.84c	52.11 ± 0.84a	82.78 ± 0.51b	17.22 ± 0.51a
	0–1	34.68	A	30.13 ± 0.64b	69.87 ± 0.64b	49.88 ± 0.68b	50.12 ± 0.68b	83.14 ± 0.43b	16.86 ± 0.42a
Y LY 900	12	41.53	A	16.96 ± 0.62a	83.04 ± 0.61b	34.82 ± 0.82a	65.23 ± 0.82b	72.71 ± 0.67a	27.32 ± 0.67c
	5	39.35	A	10.25 ± 0.44b	89.75 ± 0.44a	23.13 ± 0.82b	76.87 ± 0.82a	57.16 ± 1.00c	42.84 ± 1.00a
	0–1	36.38	A	10.96 ± 0.55b	89.04 ± 0.55a	23.93 ± 0.84b	76.07 ± 0.84a	59.84 ± 0.95b	40.16 ± 0.95b
IR 64	12	41.88	A	14.25 ± 0.51b	85.75 ± 0.56a	30.61 ± 0.69a	69.41 ± 0.69a	69.72 ± 0.55a	30.28 ± 0.55b
	5	37.83	A	15.28 ± 0.64ab	84.72 ± 0.64ab	31.32 ± 0.98a	68.68 ± 0.98a	66.54 ± 0.97b	33.46 ± 0.97a
	0–1	42.52	A	16.53 ± 0.54a	83.47 ± 0.54b	32.68 ± 0.73a	67.32 ± 0.73a	69.92 ± 0.62a	30.08 ± 0.62b

*Data are shown as the mean ± standard error of triplicate measurements. Different letters following the standard deviation denote significant differences (p < 0.05). XLY900, Xiang liangyou 900; YLY900, Y liangyou 900; EC, electrical conductivity*.

### Physicochemical Properties of Rice Starch

#### Gelatinization and Pasting Properties

Except for *T*_0_ of XLY900, salt stress increased *T*_0_, *T*_*p*_, and *T*_*C*_, and salt-sensitive cultivar IR64 was more affected (Table 3). The gelatinization enthalpy (Δ*H*) of salt-tolerant cultivars was not affected or increased under medium salt stress, but significantly decreased under high salt stress, whereas Δ*H* of salt-sensitive cultivar increased significantly under salt stress with the higher salt concentration.

**Table 3:** Effects of salt stress on starch gelatinization properties.

**Cultivars**	**EC (dS/m)**	**Salinity level**	***T_**0**_* (**°**C)**	***T_***P***_*(**°**C)**	***T_***C***_* (**°**C)**	***ΔH* (J/g)**
XLY900	12	High	72.77 ± 0.21c	82.00 ± 0.99a	87.31 ± 0.10a	3.42 ± 0.10b
	5	Medium	75.67 ± 0.33a	80.06 ± 0.47b	86.93 ± 0.44a	7.27 ± 0.08a
	0–1	Control	74.74 ± 0.34b	79.63 ± 0.24b	86.69 ± 0.72a	7.29 ± 0.41a
YLY900	12	High	75.29 ± 0.13b	82.90 ± 0.88a	88.77 ± 0.45a	2.08 ± 0.20c
	5	Medium	76.93 ± 1.07a	82.65 ± 0.43a	87.39 ± 1.19b	7.95 ± 0.39a
	0–1	Control	73.99 ± 0.66c	78.88 ± 1.03b	86.19 ± 0.44b	5.59 ± 0.86b
IR 64	12	High	76.82 ± 0.28a	82.05 ± 0.28a	87.80 ± 0.27a	6.44 ± 0.25a
	5	medium	77.64 ± 0.61a	82.10 ± 0.22a	86.95 ± 0.39a	5.75 ± 0.21b
	0–1	Control	67.75 ± 2.11b	73.46 ± 3.14b	81.96 ± 0.82b	4.68 ± 1.24c

*Data are shown as the mean ± standard error of triplicate measurements. Different letters following the standard deviation denote significant difference (p < 0.05). XLY900, Xiang liangyou 900; YLY900, Y liangyou 900; EC, electrical conductivity; T_0_, onset temperature; T_p_, peak temperature; T_c_, conclusion temperature; ΔH, enthalpy of gelatinization*.

For salt-tolerant cultivars XLY900 and YLY900, salt stress significantly decreased TV and CPV, while BD showed an increasing trend (Table 4). At the same time, it can be seen from Table 4 that salt stress reduced the PV of XLY900 and increased its SB, whereas YLY900 showed the opposite trend, which indicated that the pasting properties of XLY900 might become worse and the viscosity characteristics of YLY900 better; hence, it might be inferred that YLY900 might be more salt-tolerant than XLY900. For salt-sensitive cultivar IR64, salt stress reduced significantly PV, BD, and CPV, indicating that the pasting properties became worse. The pasting properties of different cultivars had different responses to salt stress. Rice cultivars with strong salt tolerance might have better pasting properties after salt stress treatment.

**Table 4:** Effects of salt stress on starch pasting properties.

**Cultivars**	**EC(dS/m)**	**PV**	**TV**	**BD**	**CPV**	**SB**
XLY900	12	2,047.00 ± 0.82c	1,535.00 ± 0.82c	512.00 ± 0.82a	2,713.00 ± 0.82b	1,178.00 ± 0.82a
	5	2,089.50 ± 17.90b	1,734.50 ± 40.99b	355.00 ± 23.09c	2,725.50 ± 19.05b	991.00 ± 21.94b
	0-1	2,337.00 ± 1.15a	1,857.50 ± 0.58a	479.50 ± 1.73b	2,937.50 ± 0.58a	1,080.00 ± 0.82b
YLY900	12	3,336.00 ± 80.83a	1,847.00 ± 0.82b	1,489.00 ± 80.83a	2,585.00 ± 11.55b	738.00 ± 11.55b
	5	3,422.50 ± 244.06a	2,006.25 ± 88.81a	1,416.25 ± 329.98a	2,743.75 ± 87.12a	737.50 ± 1.73b
	0–1	3153.75 ± 275.54b	2,043.25 ± 317.81a	1,110.50 ± 46.68b	2,832.25 ± 299.15a	789.00 ± 18.67a
IR64	12	2,423.25 ± 163.19b	1,601.25 ± 146.95b	822.00 ± 16.49b	2,585.50 ± 214.30b	984.25 ± 67.41a
	5	2,649.00 ± 2.31a	1,644.00 ± 17.32a	1,005.00 ± 19.63a	2,601.50 ± 4.04a	957.50 ± 13.28a
	0–1	2,651.50 ± 86.88a	1,624.75 ± 116.97a	1,026.75 ± 95.51a	2,602.50 ± 194.92a	977.75 ± 100.82a

*Data are shown as the mean ± standard error of triplicate measurements. Different letters following the standard deviation denote significant difference (p < 0.05). XLY900, Xiang liangyou 900; YLY900, Y liangyou 900; EC, electrical conductivity; PV, peak viscosity; TV, trough viscosity; CPV, final viscosity; BD, breakdown viscosity; SB, setback viscosity*.

#### Starch Crystal Characteristics

**Figure 2:**
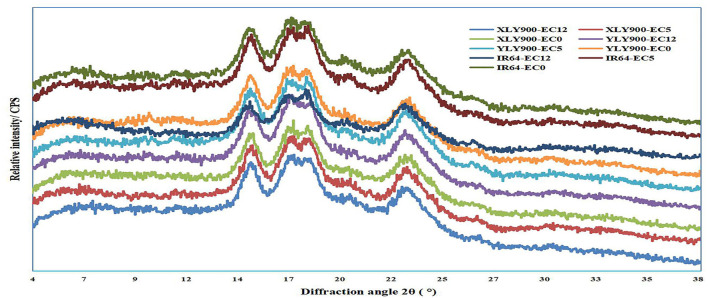
Effects of salt stress on the X-ray diffraction patterns of rice starch. XLY900, Xiang liangyou 900; YLY900, Y liangyou 900; EC12, EC5, and EC0 represent that the electrical conductivity is 12, 5, and 0–1 dS/m, respectively.

All tested starches showed similar XRD patterns, exhibiting A-type crystals with two strong 2θ peaks at ~15° and 23° and an unresolved double 2θ peak at 17° and 18°, indicating that the crystal type of rice starches was not changed by salt stress (Figure 2). Although salt stress did not change the crystal type of starch, it significantly changed the relative crystallinity of starch, indicating that salt stress destroyed the crystal structure to a certain extent (Table 2). The relative crystallinity of salt-tolerant cultivars XLY900 and YLY900 increased significantly after salt stress, while that of salt-sensitive cultivar IR64 decreased, which was mainly because the proportion of amylopectin medium and long chains in salt-tolerant cultivars increased more than that of a salt-sensitive cultivar.

## Discussion

Rice quality is an important trait for breeders to develop new rice cultivars. These traits determine directly the market price of rice and consist of a series of parameters, such as grain physical appearance, milled rice chemical composition, and structural features of rice starch. The parameters, in turn, affect the physicochemical properties of rice grains, which further reflect the ECQ of rice.

### Effects of Salt Stress on Physicochemical Properties of Rice Starch

Salt stress significantly increased the total starch content and the proportion of the medium and long chains of amylopectin but obviously decreased amylose content and the proportion of short chain of amylopectin. The decrease of amylose content might be closely related to the change in GBSSI synthase activity, while the change in total starch content was the result of the combined action of starch synthase, starch debranching enzyme, and starch branching enzyme (26, 27), indicating that GBSSI activity of XLY900 might not be affected due to its strong salt tolerance. Similarly, Thitisaksakul (14) and Cheng et al. (28) reported that changes in starch composition might be related to salt concentration levels, and low salinity might promote starch accumulation. However, in this study, it was found that high salinity might also promote starch accumulation, which might be due to the different genotypes selected. Moreover, some studies reported that peroxidase activity in salt-tolerant cultivars was higher, but lower in salt-sensitive cultivars with increased salinity levels, which might cause the inconsistency of starch content changes between salt-tolerant and salt-sensitive cultivars (29). In general, short or medium chains were lower, and long chains were higher, resulting in worse ECQ of milled rice (30). Moreover, higher protein levels prevented starch grains from absorbing water, making rice ECQ worse (31).

Changes in starch composition and structure led to the according changes in starch physicochemical properties (23). The increase of GT might be closely related to the decrease of amylose content and the proportion of short chain as well as the increase of medium and long chains of amylopectin under salt stress. The GT reflected the cooking difficulty of rice, resulting in higher cooking temperature and longer cooking time required for milled rice matured at salt stress (32). Previous studies have shown that gelatinization enthalpy was related to starch crystal structure and starch grain size distribution (33). The proportion of large starch granules was positively correlated with the gelatinization enthalpy (22, 34). The change in trends of gelatinization enthalpy of the three tested cultivars was consistent with the change in the proportion of large starch granules. The large starch granules in the salt-sensitive cultivar increased significantly, so the gelatinization enthalpy obviously increased, making gelatinization of rice more difficult.

The changes in starch granule size distribution, gelatinization enthalpy, and pasting properties under salt stress differed the trends between salt-tolerant and salt-sensitive cultivars. The surface area, volume, and number distribution of small and medium starch granules in salt-tolerant cultivars decreased first and then increased, while the large starch granules increased first and then decreased. However, the proportion of small and medium starch granules in number, volume, and surface area of salt-sensitive cultivar decreased significantly, while the proportion of large starch granules increased. The gelatinization enthalpy of salt-tolerant cultivars decreased significantly, while that of salt-sensitive cultivars increased significantly under high salt stress. The change of starch granule size distribution of salt-tolerant rice showed a certain adaptability with the increase in salt concentration, suggesting that salt-tolerant rice cultivars might start their adaptive adjustment mechanism by stimulation of certain salt concentrations to alleviate the harm caused by salt stress (35).

### Response Characteristics of Starch Physicochemical Properties to Salt Stress in Salt-Tolerant Rice Varieties

Our study found that the chalkiness of salt-tolerant cultivars increased less than that of salt-sensitive cultivars. The increase of starch accumulation was observed in salt-tolerant cultivars, while no change in salt-sensitive cultivars was observed. These results indicated that the normal grain-filling of salt-tolerant cultivars was less affected than that of salt-sensitive cultivars. A previous study has reported that salt could stimulate adenosine diphosphate-glucose pyrophosphorylase (ADPG) activity, leading further to starch accumulation (36). The starch enrichment in salt tolerance might play a key role as a chelator to chelate sodium ions to neutralize Na + toxicity (37). In contrast, high salt stress might not induce ADPG activity in semi-salt-tolerant or salt-sensitive varieties, so that starch accumulation in these rice varieties had no change or even decreased (17).

The salt-tolerant cultivars with more medium and long chains of amylopectin promoted the formation of the double helix, making the starch granule structure more stable than the salt-sensitive cultivar. Compared with salt-sensitive cultivar, salt-tolerant cultivars had lower GT and gelatinization enthalpy; therefore, their cooking quality was less affected. The viscosity characteristics of salt-tolerant cultivar with higher BD and PV as well as lower SB might become better after salt stress, while the viscosity characteristics of salt-sensitive cultivar became worse, suggesting that the integrity of the gel network might be compromised.

## Conclusion

Salt stress significantly changed the chemical composition, starch structure, and physicochemical properties of rice and ultimately led to the deterioration of ECQ of rice. Compared with salt-sensitive cultivars, the salt-tolerant cultivars exhibited lower GT and gelatinization enthalpy, better pasting properties, and more stable crystal structure; therefore, their ECQ was less affected. This study analyzed the response difference between salt-tolerant and salt-sensitive rice cultivars to medium and high salt stress, providing a reference for breeders to develop salt-tolerant rice cultivars with good cooking and eating quality.

## Data Availability Statement

The original contributions presented in the study are included in the article/Supplementary Material, further inquiries can be directed to the corresponding author.

## Author Contributions

BB: conceptualization, funding acquisition, methodology, project administration, supervision, writing, reviewing, and editing. DY and JW: conceptualization, formal analysis, investigation, visualization, methodology, and writing the original draft. QL and DZ: methodology and resources. WZ: resources and investigation. GX and QD: validation. All authors contributed to the article and approved the submitted version.

## Funding

This study was financially supported by the 2020 Research Program of Sanya Yazhou Bay Science and Technology City (SKJC-2020-02-006) and the National Natural Science Foundation of China (U19A2032).

## Conflict of Interest

QD was employed by Bio-Rice (Hunan) Co. Ltd. The remaining authors declare that the research was conducted in the absence of any commercial or financial relationships that could be construed as a potential conflict of interest.

## Publisher's Note

All claims expressed in this article are solely those of the authors and do not necessarily represent those of their affiliated organizations, or those of the publisher, the editors and the reviewers. Any product that may be evaluated in this article, or claim that may be made by its manufacturer, is not guaranteed or endorsed by the publisher.

## Supplementary Material

Supplementary Figure S1 Three cultivars were treated with salt stress at the reproductive growth stage at three salt ponds.

Supplementary Table S1 Information about the cultivars was used in the experiment.

Supplementary Table S2 Effects of salinity stress on rice milling and appearance quality.
