# Supplementary figures and images for: Effects of Salinity Stress at Reproductive Growth Stage on Rice (Oryza sativa L.) Composition, Starch Structure, and Physicochemical Properties
Source: Front Nutr. 2022 Jun 29;9:926217. doi: 10.3389/fnut.2022.926217 (PMC9277441; doi:10.3389/fnut.2022.926217)

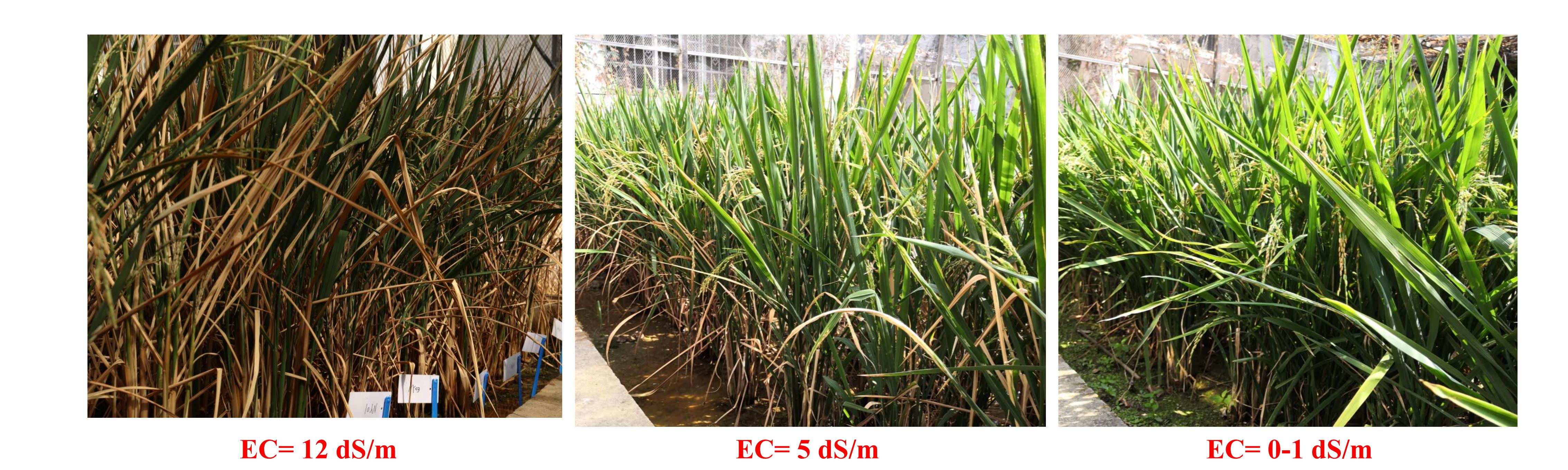

Supplement: Supplementary Figure S1 — Three cultivars were treated with salt stress at the reproductive growth stage at three salt ponds. [file Image_1.JPEG]
