# Supplementary material for: Effects of Salinity Stress at Reproductive Growth Stage on Rice (Oryza sativa L.) Composition, Starch Structure, and Physicochemical Properties
Source: Front Nutr. 2022 Jun 29;9:926217. doi: 10.3389/fnut.2022.926217 (PMC9277441; doi:10.3389/fnut.2022.926217)
Supplement: Supplementary Table S1 — Information about the cultivars was used in the experiment. [file Data_Sheet_1.docx]

**Table S1**. Information about the cultivars was used in the experiment

| Cultivars | Cultivar type | Parent source | Maturity period/days | Source institution |
| --- | --- | --- | --- | --- |
| XLY900 | Indica-type two-line hybrid rice | Guangxiang 24S(♀)  ×[R900](https://www.ricedata.cn/variety/varis/617313.htm)(♂) | 143 | Hunan Nianfeng Seed Industry Co., Ltd |
| YLY900 | Indica-type two-line hybrid rice | Y58S(♀) × R900(♂) | 141 | Biocentury Seed Industry Co., Ltd |
| IR64 | inbred indica rice | IR5657-33-2-1(♀)×  IR2061-465-1-5-5(♂) | 138 | International Rice Research Institute |

**Table S2**. Effects of salinity stress on rice milling and appearance quality

| Cultivars | EC  (dS/m) | Salinity level | Brown rice rate/% | Polished rice rate/% | Head milled rice rate/% | Chalkiness degree/% | Chalky grain rate/% | length-to-width ratio |
| --- | --- | --- | --- | --- | --- | --- | --- | --- |
| XLY900 | 12 | High | 76.37±0.56b | 68.38±0.91b | 64.72±0.85b | 18.42±0.14a | 62.00±2.65a | 2.69±0.004a |
|  | 5 | Medium | 79.98±0.05a | 72.06±0.07a | 70.56±0.47a | 12.28±0.44b | 39.67±5.86b | 2.68±0.031a |
|  | 0-1 | Control | 80.94±0.57a | 73.02±0.16a | 71.55±0.02a | 2.53±0.39c | 9.75±2.06c | 2.64±0.012a |
| YLY900 | 12 | High | 75.6±0.74b | 68.49±0.44b | 65.59±0.82b | 21.44±1.79a | 59.33±5.51a | 2.61±0.010a |
|  | 5 | Medium | 79.19±0.55a | 71.24±0.43a | 69.15±0.17a | 17.76±0.61b | 47.33±2.08b | 2.65±0.020a |
|  | 0-1 | Control | 80.01±0.71a | 72.02±1.03a | 69.92±1.96a | 2.18±0.36c | 12.25±0.96c | 2.60±0.036a |
| IR64 | 12 | High | 74.87±0.88b | 66.72±1.58b | 54.66±0.87b | 25.4±1.24a | 72.67±3.06a | 3.22±0.009a |
|  | 5 | Medium | 76.17±0.16ab | 68.56±0.35b | 64.61±2.24a | 25.28±0.91a | 74.33±4.16a | 3.20±0.017a |
|  | 0-1 | Control | 79.28±1.36a | 71.41±1.31a | 65.53±0.52a | 1.53±0.41b | 10.50±4.43b | 3.10±0.044b |

Data are shown as the mean±standard error of triplicate measurements. Different letters are followed after standard deviation to express significantly different (p < 0.05). XLY900, Xiang liangyou 900; YLY900, Y liangyou 900; EC, electrical conductivity.
